# MicroRNAs and other small RNAs in *Aedes aegypti* saliva and salivary glands following chikungunya virus infection

**DOI:** 10.1038/s41598-022-13780-3

**Published:** 2022-06-09

**Authors:** Carmine Fiorillo, Pei-Shi Yen, Alessio Colantoni, Marina Mariconti, Nayara Azevedo, Fabrizio Lombardo, Anna-Bella Failloux, Bruno Arcà

**Affiliations:** 1 Department of Public Health and Infectious Diseases – Division of Parasitology, “Sapienza” University, Piazzale Aldo Moro 5, 00185 Rome, Italy; 2 Arboviruses and Insect Vectors Unit, Institute Pasteur, 25 rue Dr. Roux, 75724 Paris Cedex 15, France; 3 Department of Biology and Biotechnology, “Sapienza” University, Piazzale Aldo Moro 5, 00185 Rome, Italy; 4 Genomics Core Facility, European Molecular Biology Laboratory, Meyerhofstrasse 1, 69117 Heidelberg, Germany

**Keywords:** Non-coding RNAs, Parasitology

## Abstract

Mosquito saliva facilitates blood feeding through the anti-haemostatic, anti-inflammatory and immunomodulatory properties of its proteins. However, the potential contribution of non-coding RNAs to host manipulation is still poorly understood. We analysed small RNAs from *Aedes aegypti* saliva and salivary glands and show here that chikungunya virus-infection triggers both the siRNA and piRNA antiviral pathways with limited effects on miRNA expression profiles. Saliva appears enriched in specific miRNA subsets and its miRNA content is well conserved among mosquitoes and ticks, clearly pointing to a non-random sorting and occurrence. Finally, we provide evidence that miRNAs from *Ae. aegypti* saliva may target human immune and inflammatory pathways, as indicated by prediction analysis and searching for experimentally validated targets of identical human miRNAs. Overall, we believe these observations convincingly support a scenario where both proteins and miRNAs from mosquito saliva are injected into vertebrates during blood feeding and contribute to the complex vector–host–pathogen interactions.

## Introduction

Mosquitoes are vectors of pathogens responsible for diseases of great public health relevance. This is the case for malaria, which is transmitted by anopheline species, as well as for several arboviral diseases transmitted by culicine mosquitoes (dengue, yellow fever, Zika virus disease, West Nile fever, chikungunya, Japanese encephalitis). Billions of people worldwide are currently at risk of mosquito-borne diseases, which may have caused over half million deaths in 2019^1,2^. Pathogen transmission typically occurs when an infected mosquito acquires the blood meal. In fact, while feeding, the mosquito vector injects the pathogen into the host skin along with saliva, a complex cocktail of bioactive compounds helping blood meal acquisition^3^. Our understanding of the complexity of blood feeding insect saliva greatly improved in the last two decades, mainly thanks to the powerful tools of transcriptomics, proteomics, and genomics^4–6^. As far as we learned, mosquito saliva carries around 100–150 salivary proteins belonging to at least 24 different families and their main physiological role is to counteract host responses to tissue injury^7–10^. First, blood vessel damage triggers the three classical components of vertebrate haemostatic response: platelet aggregation, vasoconstriction, and coagulation. In this view, it is not surprising that saliva of all blood feeding arthropods (BFAs) analysed so far carries at least one anticoagulant, one vasodilator and one inhibitor of platelet aggregation^6,11^. Furthermore, vertebrates evolved additional defence mechanisms against tissue lesions, with innate immunity and inflammation that can largely impair blood-feeding^12^. For this reason, mosquito saliva also includes proteins with anti-inflammatory and immunomodulatory properties^6,11^. Importantly, these activities can induce local modifications at the bite site, and this may affect transmission of disease agents as diverse as the malaria parasite^13–17^ and arboviruses^18–21^.

Traditionally, studies on BFA saliva have been essentially focused on salivary gland proteins. However, the recent discovery that saliva also carries microRNAs (miRNAs) pointed out that saliva of hematophagous arthropods may be a cocktail even more complex than originally anticipated^22–24^. MicroRNAs are well known for their role in post-transcriptional regulation of eucaryotic genes. They are expressed in all animal cell types, with expression patterns varying according to several variables (tissue, sex, physiological condition, etc.) and, as part of complex networks, contribute to the regulation of essentially any aspects of cell life^25,26^. MiRNAs undergo a specific processing from primary transcripts (pri-miRNAs, > 100 nt) to hairpin precursors (pre-miRNAs, ~ 80 nt) and then to mature miRNA duplexes of ~ 22 nt in length. Typically, one of the two strands of the duplex (guide strand) enters the miRNA-induced silencing complex (miRISC) and drives it to the mRNA target, promoting its degradation or translational inhibition^27^. Target recognition usually involves imperfect base pairing between the mature miRNA and the 3’UTR of the mRNA target, with the so-called seed region of the miRNA (nt 2–8) playing a crucial role in this interaction^25^. MicroRNAs are not only present within cells but also in animal body fluids^28^, where they may be embedded within exosomal microvesicles or freely circulate in complex with Argonaute (Ago) proteins or High-Density Lipoproteins (HDL)^29–31^. It is still debated whether these extracellular miRNAs play physiological roles or are just cellular byproducts^31,32^. Nevertheless, clear examples of exosomal miRNAs involvement in cell–cell communication have been provided^33–38^, and possible mechanisms of extracellular miRNAs delivery to target cells were previously described^32,39^.

Maharaj and collaborators were the first to report the presence of miRNAs in the saliva of a BFA, specifically in the culicine mosquitoes *Aedes aegypti* and *Aedes albopictus*, either uninfected or infected by the chikungunya virus (CHIKV)^40^. Afterwards, miRNAs were described in the saliva of the ticks *Ixodes ricinus*^23^ and *Haemaphysalis longicornis*^24^, and of the anopheline mosquito *Anopheles coluzzii*^22^. However, while the study on *Aedes* mosquitoes only included saliva, those on *An. coluzzii* and *I. ricinus* analysed both saliva and salivary glands. This experimental set-up allowed to reveal a differential miRNA enrichment in these two compartments, with some miRNAs more abundant in saliva, others more abundant in salivary glands and still others approximately equally distributed. These findings indicated that, at least in the mosquito *An. coluzzii* and in the tick *I. ricinus*, saliva miRNA content does not simply mirror salivary gland content, implying that some mechanism may specifically and actively convey selected miRNAs from salivary gland cells to saliva. Moreover, eleven of the most abundant miRNAs in the saliva of the malaria mosquito *An. coluzzii* were identical to human miRNAs targeting genes involved in host inflammatory and immune responses^22^. Overall, these observations suggested that anopheline mosquitoes, during blood feeding, inject into the host skin both salivary proteins and miRNAs, which may be acting in concert to manipulate host inflammatory and immune responses. It is likely that a similar saliva-specific enrichment is also found in *Aedes* mosquitoes, although this cannot be taken for granted considering that anophelines and culicines diverged around 120–150 million years ago^41^. The pioneering investigation on saliva miRNAs of *Ae. aegypti* and *Ae. albopictus*^40^ was focused on saliva of uninfected and CHIKV-infected mosquitoes, but did not include either salivary gland samples or replicates. This experimental design precluded any comparison between saliva and salivary glands and could not provide statistical support to the difference observed between uninfected and CHIKV-infected mosquitoes. To verify whether previous observations on saliva miRNAs made in *Anopheles* were also valid in *Aedes*, we analysed triplicate small RNA samples from saliva and salivary glands of uninfected or CHIKV-infected *Ae. aegypti*. The results reported here provide sound experimental evidence that selected miRNA subsets are preferentially accumulated in mosquito saliva and may contribute, along with salivary proteins, to vertebrate host manipulation with potential implications for the transmission of pathogens. Moreover, the analysis of reads mapping to the CHIKV genome and antigenome indicated that CHIKV infection triggers in *Ae. aegypti* salivary glands both the siRNA and piRNA pathways, an organ-specific antiviral response not previously reported in *Aedes* mosquitoes.

## Results

### Deep sequencing and mapping to the *Aedes aegypti* genome

Small RNAs were extracted from saliva and salivary glands of both uninfected and CHIKV-infected *Ae. aegypti*. Three biological replicates from uninfected saliva (S), uninfected salivary glands (G), infected saliva (SCK) and infected salivary glands (GCK) were used for small RNA libraries construction and Illumina high-throughput sequencing. Overall, RNA-seq yielded ~ 275 million raw reads (S = 57.45; G = 60.80; SCK = 36.91; GCK = 119.32). After adapter trimming, quality filtering and size selection ~ 232 million reads (MR) were retained, with a total of ~ 150 MR aligning to the *Ae. aegypti* genome (S = 12.91; G = 42.40; SCK = 11.84; GCK = 82.86; Table 1). Reads representing ribosomal RNAs were subtracted. The remaining were aligned to a list including 327 *Ae. aegypti* miRNA precursors plus other ncRNAs (see Methods section and Supplementary file S1) and the mapping reads were used to investigate the linear relationships between replicates of the different samples. The correlation between replicates was high for all samples (Spearman’s correlation coefficients: S, 0.88–0.89; G, 0.85–0.90; SCK, 0.77–0.82; GCK, 0.91–0.95; Supplementary Figure S1A-B). Variation between libraries was evaluated calculating distances based on fold change and biological coefficient of variation (Supplementary Figure S1C-D). Salivary gland samples (G and GCK) clustered closely together and independently from the saliva samples (S and SCK), with SCK2 being more distant from the other saliva samples.

**Table 1:** Small RNA deep sequencing and mapping.

	Raw reads	Filtered	AaegL5	CHIKV (+)	CHIKV (−)
S	57.45	48.75	12.91	4.39 × 10^–4^	3.67 × 10^–4^
G	60.80	49.86	42.40	16.36 × 10^–4^	13.71 × 10^–4^
SCK	36.91	31.70	11.84	0.11	0.06
GCK	119.32	102.31	82.86	3.03	2.56
Total	274.48	232.62	150.01	3.14	2.63

Numbers indicate million reads. Filtered, reads remaining after adapter removal and size selection (≥ 14). Reads mapping to the *Ae. aegypti* genome (AaegL5) and to the chikungunya virus (CHIKV) genome (+) and antigenome (−) are indicated.

**Figure 1:**
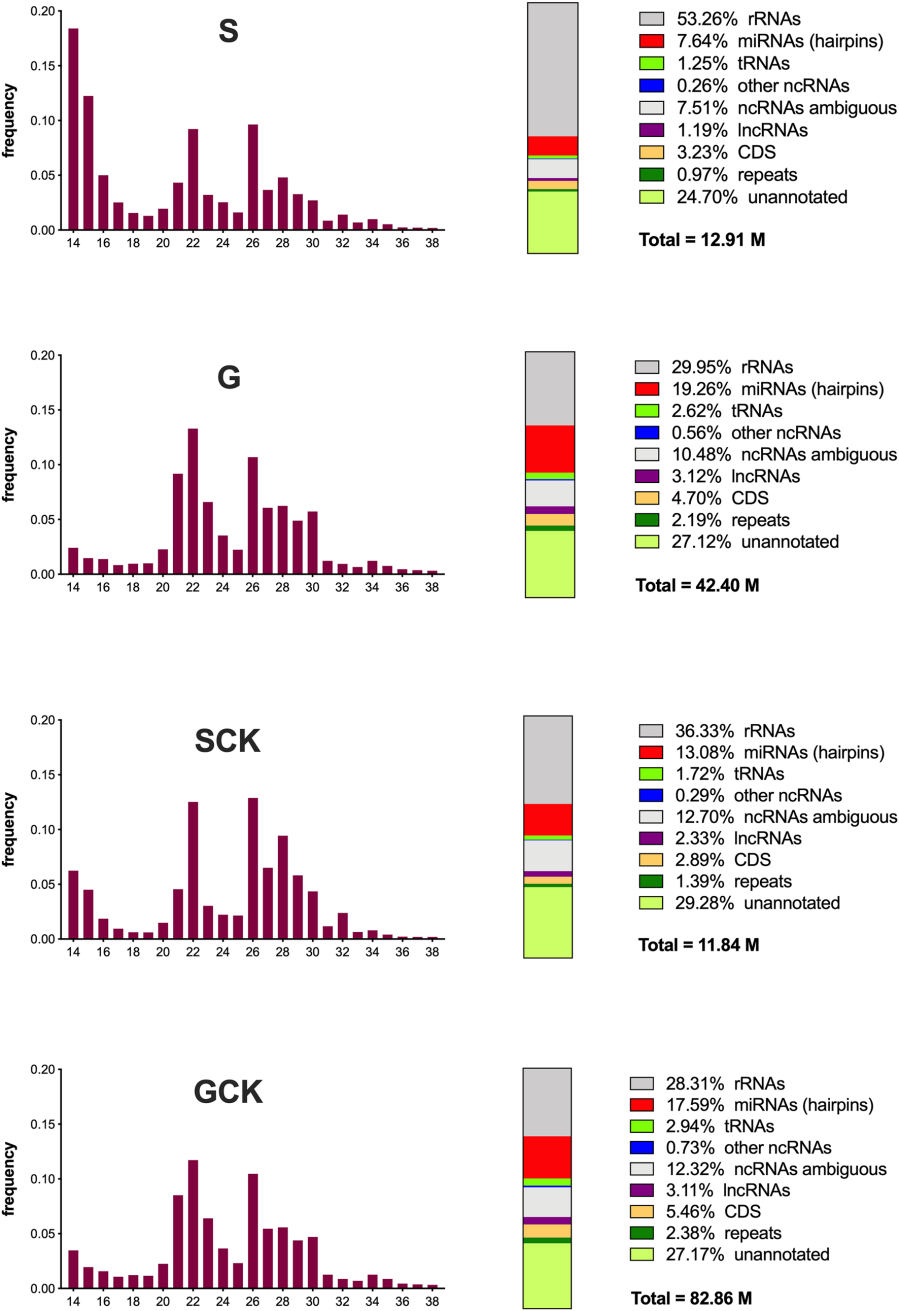
Features of small RNAs sequenced from the four *Aedes aegypti* samples. The bar plots on the left show the size distribution of reads 14–38 nt in length (expressed as percentage) mapping to the *Ae. aegypti* genome (AaegL5) and depleted of those aligning to rRNAs. The vertical slices on the right summarize the results of read alignment to *Ae. aegypti* rRNAs, miRNA precursors, tRNAs, other short non-coding RNAs (including snoRNAs, snRNAs, etc.), long non-coding RNAs, coding transcripts and repeats. Numbers indicate percentage of reads on the total in each sample. ncRNAs ambiguous, reads not assigned to a unique ncRNA entry because of multimapping (mostly aligning to tRNAs). Unannotated, reads mapping to AaegL5 but no to the other classes. Total, number of reads mapping to AaegL5. Percentage of reads mapping to miRNA precursors in the different samples, as described in the text, were compared by the Chi-square test with Yates’ correction.

The proportion of reads aligning to the different classes of RNAs and to unannotated regions of the *Ae. aegypti* genome in the four samples are shown as color-coded vertical slices in Fig. 1. Variation in the relative abundance of reads mapping to rRNAs and miRNA precursors was especially evident, whereas reads mapping to other classes of ncRNAs as well as to coding sequences, repeats and to unannotated regions of the genome were roughly comparable in the different samples. The proportion of reads mapping to rRNAs was higher in the saliva samples (S, 53.3%; SCK, 36.3%) as compared to salivary gland samples (G, 29.9%; GCK, 28.3%). This is most likely due to partial degradation of large rRNAs occurring during the elaborated saliva collection procedure, and a similar observation was made in a previous study on the mosquito *An. coluzzii*^22^. The percentage of reads mapping to miRNA hairpins was, not surprisingly, significantly higher in the salivary gland than in the saliva samples, both in uninfected and in CHIKV-infected mosquitoes (*p* < 0.0001). Interestingly, following CHIKV infection the proportion of reads mapping to miRNA precursors significantly increased in saliva (S = 7.6% vs SCK = 13.1%, *p* < 0.0001) whereas, on the contrary, showed a slight decrease in salivary glands (G = 19.3% vs GCK = 17.6%, *p* < 0.0001), suggesting that viral infection may increase the number of miRNAs released from salivary gland cells into saliva.

The size distribution of reads mapping to the *Ae. aegypti* genome and subtracted of rRNAs is also shown in Fig. 1. All four samples showed two clear peaks at 22 nt and 26 nt and a third, less pronounced and broader peak, around 28 nt. The peaks at 21–23 nt (size expected by most mature miRNAs) and at 27–30 nt (usually interpreted as possibly due to piRNAs) are commonly found in small RNA-seq studies in mosquitoes. On the contrary the 26 nt peak is unusual and, surprisingly, it was mainly represented (62.8–77.2%) by a tRNA-derived fragment (tRF), more precisely by the 5’end of the mature Gly-GCC tRNA (Gly-GCC 5tRF, 5’-GCATCGGTGGTTCAGTGGTAGAATGC-3’). In the past few years tRNA fragments emerged as a new class of short ncRNAs with specific biological functions^42^. The significance and possible role of this 26 nt Gly-GCC 5tRF in *Ae. aegypti* physiology is presently unknown; however, it is worth pointing out that it represented the most abundant ncRNA across the 12 libraries sequenced here (globally around 8 MR, i.e., 5.3% of the reads mapping to the *Ae. aegypti* genome). An additional peak at 14–15 nt was present in the S sample, most likely as result of some partial RNA degradation (see above).

### Activation of siRNA and piRNA antiviral pathways following chikungunya virus infection

**Figure 2:**
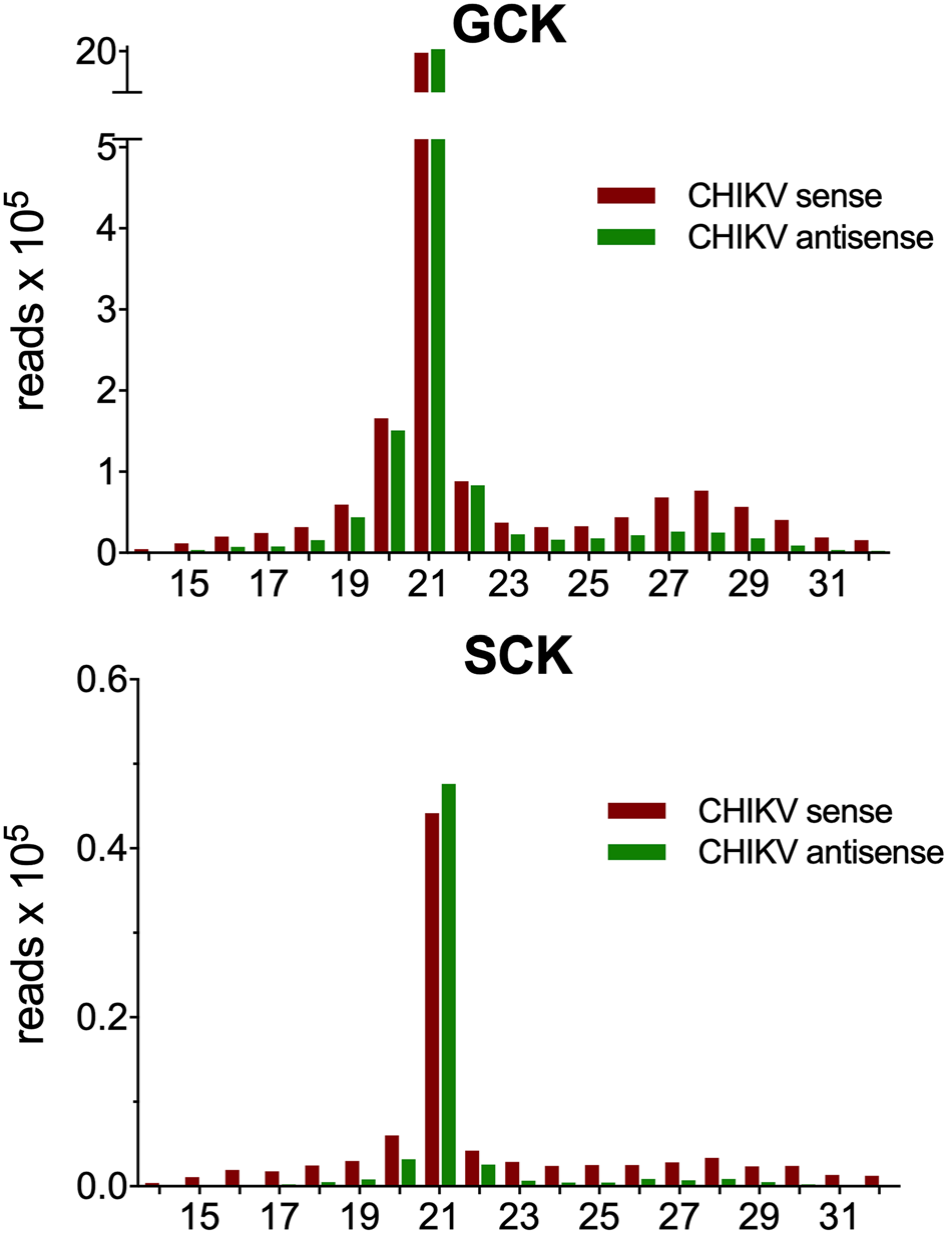
Size distribution of CHIKV-specific small RNAs in infected *Ae. aegypti* salivary glands and saliva. The number of reads mapping to the CHIKV genome (red) and antigenome (green) are reported on the Y-axis whereas the X-axis indicates the length in nucleotides of the small RNAs. GCK, CHIKV-infected salivary glands; SCK, CHIKV-infected saliva.

It is known that in response to arboviral infection *Aedes* mosquitoes mount an anti-viral immune response that involves several innate immune pathways, including the activation of the short interfering RNA (siRNA) and Piwi-interacting RNA (piRNA) pathways^43–45^. The siRNA pathway is triggered by the recognition of long double-stranded RNA (dsRNA) of viral origin by Dicer 2 (Dcr2), and involves the production of virus-specific siRNAs (vsiRNAs) of 21 nt. The piRNA pathway is initiated by viral single strand RNA (ssRNA) and leads to the production of 23–30 nt long piRNA-like small RNAs of viral origin (vpiRNAs), which originate by a specific ping-pong amplification mechanism^43–46^. To shed some light on the small RNA-mediated antiviral response in the salivary glands of *Aedes* mosquitoes, which has not been investigated so far, we aligned those reads not mapping to the AaegL5 genome assembly to the CHIKV genome (Reunion, strain 06–21). Only a few hundred reads from uninfected samples mapped to the CHIKV genome (S = 439; G = 1,636); on the contrary, infected saliva and salivary glands yielded from one hundred thousand to three million mapping reads, respectively (SCK = 106,066; CGK = 3,030,548). A similar result was obtained by alignment to the CHIKV antigenome, with a significant number of mapping reads only found in the infected samples (S = 367; G = 1,371; SCK = 60,990; GCK = 2,565,823; Table 1). Fractionation according to size of reads mapping to CHIKV genome and antigenome yielded a prominent peak at 21 nt in both GCK and SCK samples (Fig. 2), a size fully consistent with recognition and cutting of long dsRNA of viral origin by Dcr2^43,46^. A broad and much smaller peak, in the range of 26–30 nt and preferentially derived from the virus (+) strand was also visible. When we looked in more detail at the composition of the 28 nt fraction in the GCK sample (i.e. the most represented fraction in the most abundant sample) we found the typical signatures of the ping-pong amplification mechanism^43,44,46^: (i) A10 bias, that is enrichment for A at position 10 in the reads mapping to the sense strand (76,780 total reads, A10 = 61.6%); (ii) U1 bias, that is enrichment for U at position 1 in the reads mapping to the antisense strand (25,479 total reads, U1 = 80.8%) (Supplementary Figure S2). Similar results were obtained considering the 26–30 nt fractions rather than the single 28 nt fraction [(+) strand 286,841 total reads, A10 = 57.6%; (−) strand 100,822 total reads, U1 = 79.1%]. Overall, these observations strongly suggest that infection of *Ae. aegypti* salivary glands by the CHIKV triggers the siRNA pathway, with abundant production of CHIKV-specific vsiRNAs, and induces, although to a much smaller extent, the piRNA pathway with production of CHIKV-specific vpiRNAs.

### miRNAs in uninfected and chikungunya-infected saliva and salivary glands

**Figure 3:**
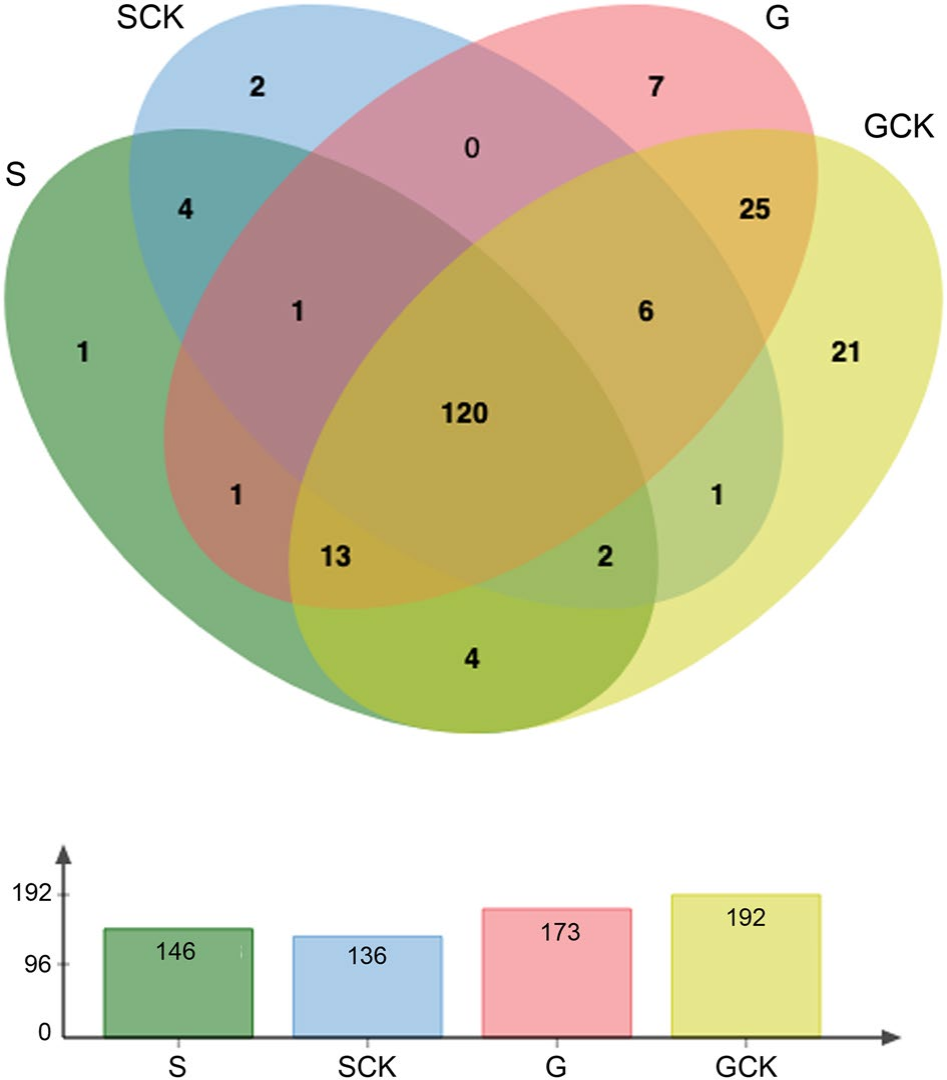
Distribution of the 208 mature miRNAs found in *Aedes aegypti* saliva and salivary glands. The Venn diagram shows the degree of overlap between miRNAs expressed in the four samples. S (green) and SCK (light blue), saliva from uninfected and CHIKV-infected mosquitoes; G (pink) and GCK (yellow), salivary glands from uninfected and CHIKV-infected mosquitoes. The number of miRNAs found expressed in each sample is shown below the diagram. Venn diagram obtained by the jvenn tool at http://jvenn.toulouse.inra.fr/app/example.html.

Overall, considering a miRNA as expressed in each sample when having read counts in at least two of the three replicates and a mean Counts Per Million (CPM) ≥ 3.0, we found in silico evidence for the expression of 208 miRNAs (Supplementary File S2). Not surprisingly, the number of miRNAs was lower in saliva as compared to salivary glands (S = 146, G = 173; SCK = 136, GCK, 192), with the 50 most abundant miRNAs in each sample being supported by over 1000 mean CPM. One hundred-twenty miRNAs were common among the four samples, 22 to three samples, 35 to two samples whereas a total of 31 were unique to S (1), SCK (2), G (7) or GCK (21) (Fig. 3). These sample-specific miRNAs were of low abundance, with mean CPM values around or below 10 (Supplementary File S2); only exception was the S-specific novelMiR-12819 which, in any case, ranked only 92nd over 146 total miRNAs expressed in uninfected saliva. The very low level of expression of these sample-specific miRNAs discouraged any search for potential targets either in the mosquito or in the human host.

Among the 208 miRNAs expressed in our samples, 45 were putative novel *Ae. aegypti* miRNAs predicted by the miRDeep* tool. To verify whether any of these 45 predicted miRNAs matched previously known arthropod miRNAs, we searched miRBase using as query both mature and precursors. Only novelMiR-10576 showed a significant identity to a known miRNA, specifically aae-miR-11921 (mature 95% identity, 90% coverage; hairpin 88% identity, 87% coverage). Noteworthy, the large majority of these 45 putative novel miRNAs were of relatively low abundance, with only 10 miRNAs being supported by > 50 CPM per library and 12 miRNAs represented in more than two thirds of the libraries (Supplementary Figure S3). These predicted miRNAs were marginally represented in the saliva samples and the two most abundant (novelMiR-13799 and novelMiR-12744) ranked from 48 to 56th in the S and SCK samples. Considering the low levels of these putative miRNAs in the saliva samples and taking into account that the discovery of novel *Ae. aegypti* miRNAs was by itself beyond the scope of our study, we did not undertake any experimental validation. Nevertheless, a list of these predicted miRNAs, their sequence and genomic location, as well as a few additional potentially useful information are provided in Supplementary File S3.

### Differential miRNA expression in salivary glands versus saliva and upon viral infection

**Figure 4:**
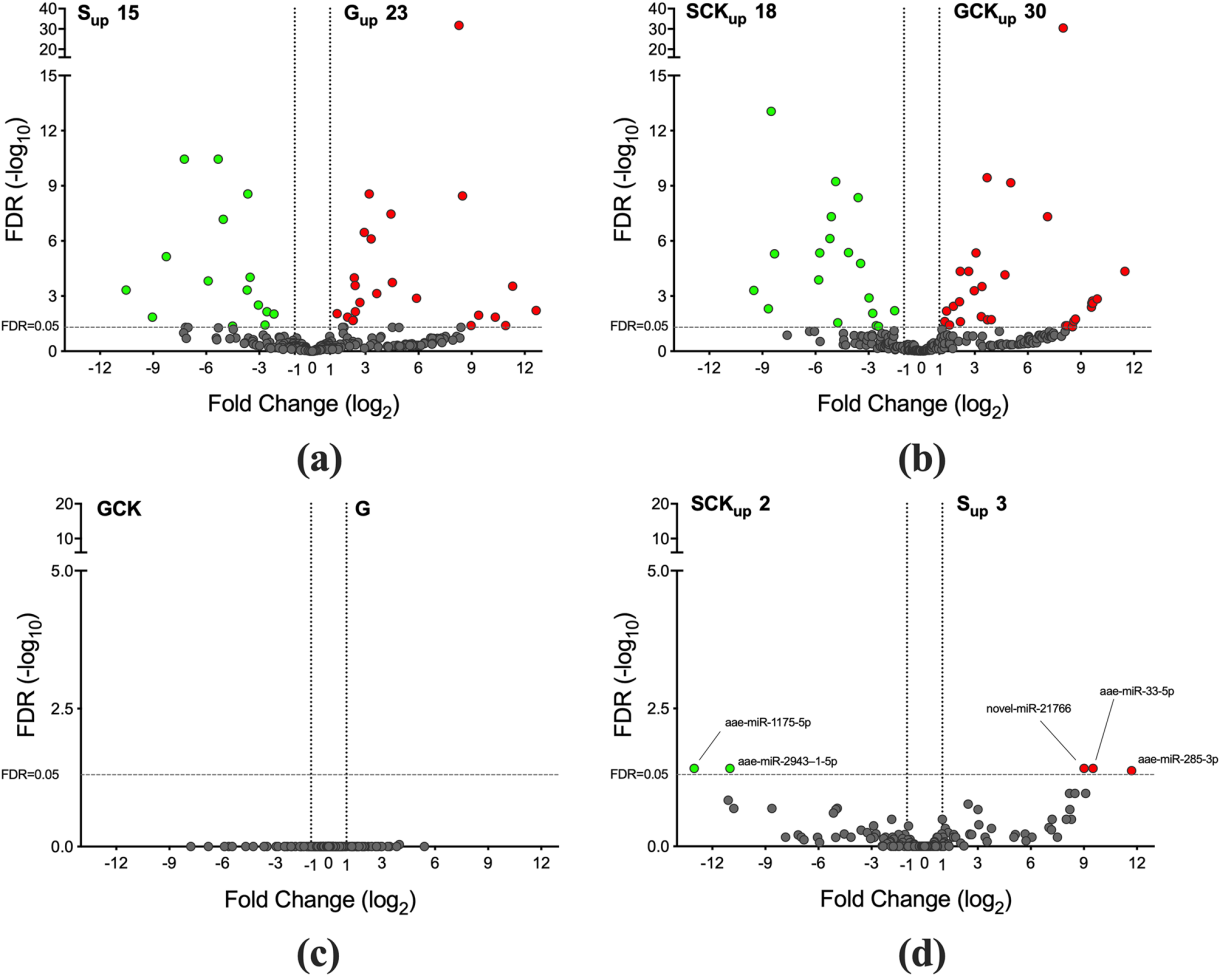
Differential abundance of miRNAs in uninfected and CHIKV-infected *Aedes aegypti* saliva and salivary glands. The volcano plots show the differential abundance of miRNAs in the following pairwise comparisons: (**a**) S-G; (**b**) SCK-GCK; (**c**) GCK-G; (**d**) SCK-S. The log2 of fold change (FC) versus the negative log10 of false discovery rate (FDR) as calculated by edgeR are reported. Vertical dotted lines mark |log2(FC)|= 1, the horizontal dashed line marks FDR = 0.05. miRNAs with a |log2(FC)| > 1 and FDR < 0.05 in the different pairwise comparisons are shown either in green or red.

Reads mapping to mature miRNAs in the different samples were used to assess differential expression using the edgeR software package^47,48^. Clusters of miRNAs with different expression profiles were clearly visible in the expression heatmap, especially when comparing salivary glands to saliva samples (Supplementary Figure S4). More specifically, pairwise comparison between saliva and salivary glands in uninfected mosquitoes revealed a significantly higher abundance of 15 miRNAs in saliva and 23 miRNAs in salivary glands (Fig. 4a). A similar result was obtained in CHIKV-infected *Ae. aegypti* (Fig. 4b), with 18 miRNAs significantly enriched in SCK and 30 miRNAs overexpressed in the GCK sample. Notably, independently from the infection status, saliva samples shared 14 up-regulated miRNAs (out of 15), whereas a more limited overlap (17 out of 23 miRNAs) was found between uninfected (G) and infected (GCK) salivary glands. The lists of miRNAs differentially expressed (|log2(FC)| > 1, FDR < 0.05) in the different pairwise comparisons are provided in Supplementary File S4 along with relative counts and CPM in the different replicates/samples.

Surprisingly, no significant difference was found between uninfected (G) and infected (GCK) salivary glands (Fig. 4c). In fact, even though several miRNAs showed a |log2(FC)| > 1 (34 with FC ≤ 0.5, 36 with FC ≥ 2.0), only for 8 of them the *p* value was lower than 0.05 and, in all cases, false discovery rates (FDRs) were very high (> 0.92). A few differences were observed comparing the saliva samples S and SCK: three miRNAs appeared more abundant in uninfected saliva (aae-miR-33-5p, aae-miR-285-3p, novelMiR-21766) and two in infected saliva (aae-miR-1175-5p, aae-miR-2943-1-5p; Fig. 4d). However, FDR values (range 0.038–0.043) were close to the threshold for significance (usually set to 0.05), indicating a rather weak statistical support. Moreover, and more importantly, a careful data analysis revealed that the number of reads mapping to these 5 miRNAs was either not consistent between replicates or in most cases zero (Supplementary File S4, worksheet SvsSCK), suggesting as not very likely that these miRNAs may play any biological role. From these observations we conclude that, at least in our experimental conditions, infection by the chikungunya virus does not significantly modulate miRNA expression profiles in *Ae. aegypti* saliva and salivary glands.

### Asymmetric miRNA distribution between saliva and salivary glands

**Figure 5:**
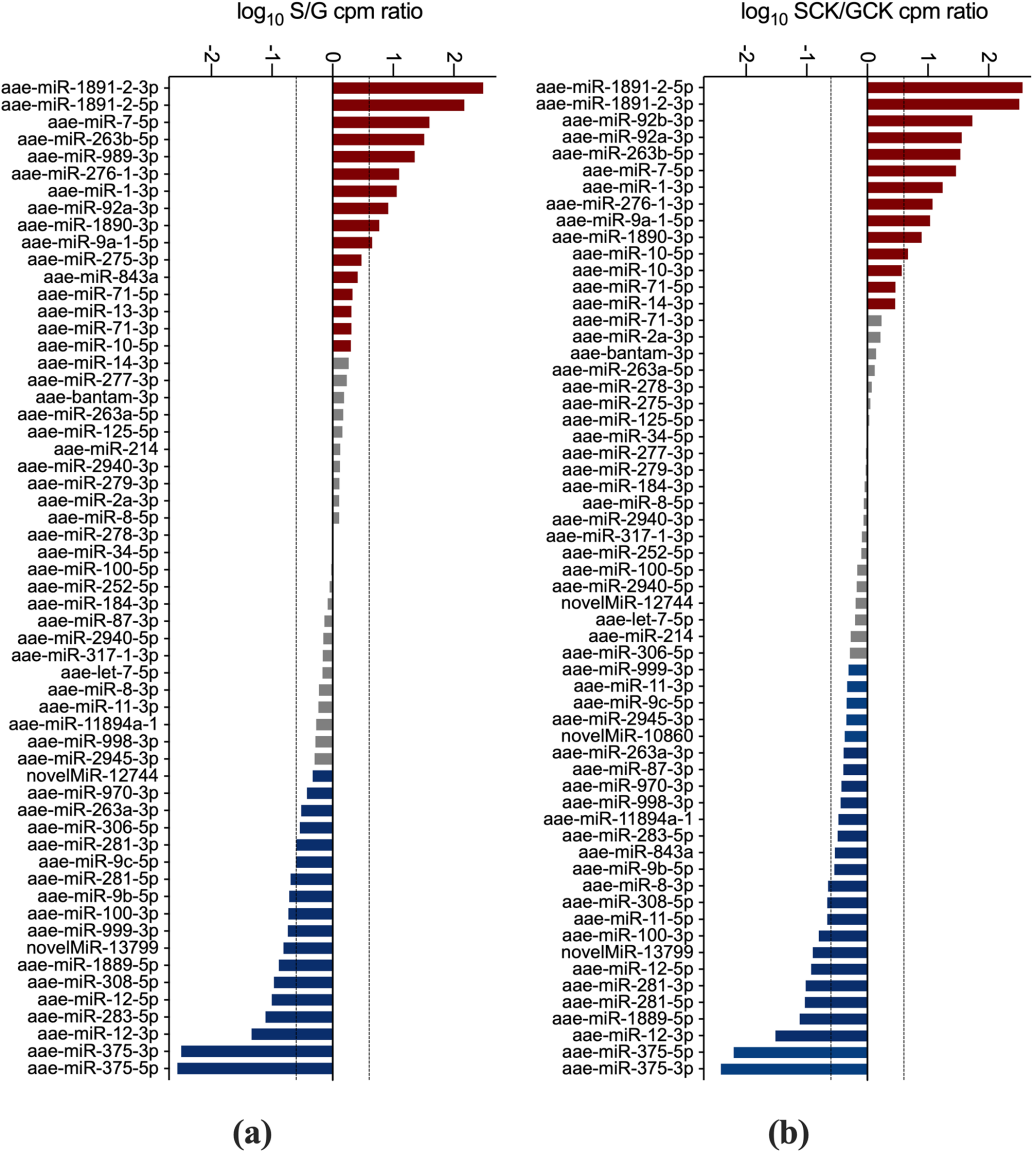
Asymmetric distribution of miRNAs in saliva and salivary glands of uninfected and CHIKV-infected *Ae. aegypti*. Comparison of the mean CPM values of the 50 most abundant miRNAs in saliva and salivary glands of uninfected and chikungunya-infected mosquitoes. The log10 of the S/G (**a**) and SCK/GCK (**b**) mean CPM ratios are reported. MicroRNAs with a ratio ≥ 2.0 are shown in red, those with a ratio ≤ 0.5 in blue and miRNAs with ratios > 0.5 and < 2.0 in grey. Dashed lines mark the limits of 4-fold overexpression.

According to differential expression analysis, subsets of specific miRNAs appeared unequally distributed between salivary glands and saliva, both in uninfected and in infected mosquitoes. This asymmetric distribution was even more strikingly evident when the mean CPM values of the 50 most abundant miRNAs in salivary glands and saliva were compared. Notably, these miRNAs were all well represented in the different libraries, as indicated by the range of their mean CPM values in the four samples: S (1033–188,246), SCK (1228–241,459), G (1235–101,763), GCK (1109–83,711). Considering a |log2(FC)| > 1 as threshold, we found that some selected groups of miRNAs were preferentially directed toward secretion into saliva, some were preferentially retained into salivary gland cells and some others were evenly distributed between the two samples/compartments (Fig. 5). This differential allocation was independent from the infection status, and most of the selectively enriched miRNAs were present in the saliva (11/16) or salivary glands (16/18) of both uninfected and CHIKV-infected *Ae. aegypti* mosquitoes. These observations point to the existence of some specific sorting mechanism acting on selected subsets of miRNAs and determining their specific enrichment in mosquito saliva or in salivary gland cells.

### Comparison with miRNAs from saliva of other mosquitoes and beyond

In a previous small RNA-seq study on *Ae. aegypti* saliva, a total of 103 miRNAs were identified^40^. Considering both uninfected and CHIKV-infected mosquitoes, we found a total of 136 known plus 19 putative novel mature miRNAs, with an extension of the *Ae. aegypti* saliva miRNA catalogue of at least 30%. Overall, there was a good concordance among the two studies, with the large majority of the most abundant miRNAs in our saliva samples (92–94% among the top 50) also present in the other list. This quite good concordance between independent studies strengthens the idea that selected subset of miRNAs are specifically conveyed toward *Aedes* saliva. Interestingly, we previously found that several miRNAs abundant in *An. coluzzii* saliva were identical to human miRNAs targeting host genes involved in immune and/or inflammatory responses^22^. To verify whether this was the case for *Ae. aegypti* too, we searched miRBase and found that 11 out of the top 30 saliva miRNAs from uninfected mosquitoes were essentially identical to human miRNAs (Table 2). The number was slightly higher in the saliva of CHIKV-infected *Ae. aegypti*, where two additional miRNAs identical to human miRNAs (miR-10-5p and miR-92a-3p) were found. These two miRNAs were also present in the saliva of uninfected mosquitoes (33rd and 40th) and, intriguingly, all these 13 miRNAs were also among the top 30 in the saliva of *An. coluzzii*^22^.

**Table 2:** Human orthologues among the 30 most abundant saliva miRNAs from uninfected *Ae. aegypti*.

*Ae. aegypti*	sequence	*H. sapiens*	seed	mm	alignment
aae-miR-14-3p	TCAGTCTTTTTCTCTCTCCTAT	–	–	–	–
aae-miR-1891-2-5p	TGAGGAGTTAATTTGCGTGTTT	–	–	–	–
**aae-miR-1-3p**	TGGAATGTAAAGAAGTATGGAG	hsa-miR-1-3p	Y	1	1–22/1–21
aae-miR-276-1-3p	TAGGAACTTCATACCGTGCTC	–	–	–	–
**aae-miR-263a-5p**	AATGGCACTGGAAGAATTCACGG	hsa-miR-183-5p	Y	1	2–21/2–21
**aae-miR-7-5p**	TGGAAGACTAGTGATTTTGTTGT	hsa-miR-7-5p	Y	-	1–23/1–23
**aae-miR-34-5p**	TGGCAGTGTGGTTAGCTGGTTG	hsa-miR-34c-5p	Y	2	2–22/2–22
aae-miR-2940-3p	GTCGACAGGGAGATAAATCACT	–	–	–	–
aae-miR-317-1-3p	TGAACACAGCTGGTGGTATCT	–	–	–	–
**aae-let-7-5p**	TGAGGTAGTTGGTTGTATAGT	hsa-let-7a-5p	Y	1	1–21/1–21
aae-miR-263b-5p	CTTGGCACTGGGAGAATTCACAG	–	–	–	–
aae-miR-2940-5p	TGGTTTATCTTATCTGTCGAGGC	–	–	–	–
**aae-miR-8-3p**	TAATACTGTCAGGTAAAGATGTC	hsa-miR-141-3p	Y/N	2	1–21/1–21
**aae-miR-184-3p**	TGGACGGAGAACTGATAAGGGC	hsa-miR-184	Y	–	–
aae-miR-281-3p	TGTCATGGAATTGCTCTCTTTA	–	–	–	–
**aae-miR-100-5p**	AACCCGTAGATCCGAACTTGTG	hsa-miR-100-5p	Y	-	1–22/1–22
aae-miR-281-5p	AAGAGAGCTATCCGTCGAC	–	–	–	–
aae-miR-279-3p	TGACTAGATCCACACTCATTAA	–	–	–	–
aae-miR-277-3p	TAAATGCACTATCTGGTACGAC	–	–	–	–
aae-bantam-3p	TGAGATCATTTTGAAAGCTGAT	–	–	–	–
aae-miR-87-3p	GTGAGCAAATTTTCAGGTGTGT	–	–	–	–
aae-miR-843a	GTCCTGTCACGGTCGCCA	–	–	–	–
aae-miR-970-3p	TCATAAGACACACGCGGCTAT	–	–	–	–
**aae-miR-8-5p**	CATCTTACCGGGCAGCATTAGA	hsa-miR-200b-5p	Y	2	1–22/1–22
**aae-miR-9a-1-5p**	TCTTTGGTTATCTAGCTGTATGA	hsa-miR-9-5p	Y	–	1–23/1–23
**aae-miR-125-5p**	TCCCTGAGACCCTAACTTGTGA	hsa-miR-125b-5p	Y	–	1–22/1–22
aae-miR-1889-5p	TAATCTCAAATTGTAACAGTGG	–	–	–	–
aae-miR-2945-3p	TGACTAGAGGCAGACTCGTTTA	–	–	–	–
aae-miR-252-5p	TAAGTACTAGTGCCGCAGGAG	–	–	–	–
aae-miR-275-3p	TCAGGTACCTGAAGTAGCGC	–	–	–	–

Y, seed (nt 2–8) fully conserved; Y/N, seed partially conserved; mm, total number of mismatches in the aligned region. *Aedes aegypti* miRNAs with human orthologues are in bold.

We also verified whether the top 30 miRNAs from *Ae. aegypti* saliva were present among the top 50: (I) in human^49^ saliva; (II) in saliva of the mosquitoes *An. coluzzii*^22^, *Ae. aegypti*^40^ and *Ae. albopictus*^40^; (III) in saliva of the ticks *I. ricinus*^23^ and *H. longicornis*^24^; IV) in exosomes released from the parasitic nematodes *Brugia malayi*^38^ and *Heligmosomoides polygyrus*^33^. *Aedes aegypti* mosquitoes shared 24 miRNAs with *An. coluzzii*, 22 with *Ae. albopictus*, 16 and 10 with the distantly related ticks *I. ricinus* and *H. longicornis*. Finally, 12 miRNAs were homologous to those found into exosomes secreted by *B. malayi* and *H. polygyrus*, two parasitic nematodes that establish long-term relationships with their vertebrate hosts (Table 3). Surprisingly, even though 11 of the top 30 miRNAs in *Ae. aegypti* saliva were identical to human miRNAs, only one was also found among the top 50 in human saliva. On the contrary, this specific miRNA subset was highly conserved in the saliva of BFAs. Full conservation (11/11) was found in *An. coluzzi,* and the vast majority (7–8 out of 11) was also present in the saliva of the ticks *I. ricinus* and *H. longicornis*. Maharaj and colleagues, in their study on the saliva of *Ae. aegypti* and *Ae. albopictus*, did not find three of these miRNAs (miR-1-3p, miR-7-5p and miR-9a-1-5p), perhaps because of the lower sequencing depth of this study^40^. Intriguingly, 6 to 8 miRNAs mimicking human miRNAs were also present in exosomes secreted by parasitic nematodes and suggested to be involved in manipulation of host responses^33,38^.

**Table 3:** Distribution in other species of *Aedes aegypti* saliva miRNAs found in this study.

*Aedes aegypti*	*Homo sapiens*	*Anopheles coluzzii*	*Aedes aegypti*	*Aedes albopictus*	*Ixodes ricinus*	*Haemaph. longicornis*	*Brugia malayi*	*Heligmos. polygyrus*
**aae-miR-14-3p**		**√**	**√**	**√**				
**aae-miR-1891-2-5p**		**√**	**√**	**√**				
aae-miR-1-3p		**√**			**√**	**√**	**√**	**√**
**aae-miR-276-1-3p**		**√**	**√**	**√**	**√**	**√**		
aae-miR-263a-5p		**√**	**√**	**√**	**√**	**√**		**√**
aae-miR-7-5p		**√**			**√**	**√**	**√**	
aae-miR-34-5p		**√**	**√**	**√**			**√**	**√**
**aae-miR-2940-3p**			**√**	**√**				
**aae-miR-317-1-3p**		**√**	**√**	**√**	**√**			
aae-let-7-5p	**√**	**√**	**√**	**√**	**√**	**√**	**√**	**√**
**aae-miR-263b-5p**		**√**						
**aae-miR-2940-5p**			**√**	**√**				
aae-miR-8-3p		**√**	**√**	**√**	**√**			**√**
aae-miR-184-3p		**√**	**√**	**√**		**√**		
**aae-miR-281-3p**		**√**	**√**	**√**			**√**	
aae-miR-100-5p		**√**	**√**	**√**	**√**	**√**	**√**	**√**
**aae-miR-281-5p**		**√**	**√**	**√**	**√**		**√**	
**aae-miR-279-3p**		**√**	**√**	**√**	**√**		**√**	**√**
**aae-miR-277-3p**		**√**	**√**	**√**				
**aae-bantam-3p**		**√**	**√**	**√**			**√**	**√**
**aae-miR-87-3p**			**√**			**√**	**√**	**√**
**aae-miR-843a**								
**aae-miR-970-3p**			**√**	**√**				
aae-miR-8-5p		**√**	**√**	**√**	**√**			
aae-miR-9a-1-5p		**√**			**√**	**√**	**√**	**√**
aae-miR-125-5p		**√**	**√**	**√**	**√**			**√**
**aae-miR-1889-5p**			**√**		**√**			
**aae-miR-2945-3p**		**√**						**√**
**aae-miR-252-5p**		**√**	**√**	**√**	**√**		**√**	
**aae-miR-275-3p**		**√**	**√**	**√**	**√**	**√**		
Total	**1**	**24**	**24**	**22**	**16**	**10**	**12**	**12**
Mimicking human		**11**	**8**	**8**	**8**	**7**	**6**	**8**

MicroRNAs homologous to the top 30 miRNAs found in the saliva of *Aedes aegypti* were searched among the top 50 in human saliva (Yeri et al., 2017), in the saliva of *Anopheles coluzzii* (Arcà et al., 2019), *Aedes aegypti* and *Aedes albopictus* (Maharaj et al., 2015), *Ixodes ricinus* (Hackenberg et al., 2017), *Haemaphysalis longicornis* (Malik et al., 2019) and among the top 50 exosomal miRNAs from *Brugia malayi* (Zamanian et al., 2015) and *Heligmosomoides polygyrus* (Buck et al., 2014). The total number of conserved miRNAs and of miRNA identical to human miRNAs are shown at the bottom. *Aedes aegypti* miRNAs identical (or almost identical) to human miRNAs are highlighted in bold.

### Prediction of putative saliva miRNAs targets

To get insights into potential host mRNA targets at the biting site, we took advantage of the miRNAconsTarget tool and the prediction programs TargetSpy^50^, miRanda^51^ and PITA^52^. To improve prediction specificity, two groups of four *Ae. aegypti* miRNAs, a saliva set and a midgut control set, were used as query. The saliva miRNAs included aae-miR-14-3p, aae-miR-1891-2-5p, aae-miR-1-3p and aae-miR-276-1-3p; these miRNAs, independently from the infection status, were the four most abundant in *Ae. aegypti* saliva and significantly enriched as compared to salivary glands. The control miRNAs list included the four miRNAs most abundantly expressed in *Ae. aegypti* midgut (aae-miR-281-3p, aae-miR-100-5p, aae-miR-184-3p and aae-miR-283-5p) and was obtained retrieving from SRA a small RNA dataset deposited by Sinclair and colleagues^53^. These miRNAs were also present in our saliva samples but at lower abundance and belonged to the salivary gland enriched or equally distributed category of miRNAs reported in Fig. 5. As targets for prediction, we used a “skin set”, made up of 15,668 3′UTRs from transcripts expressed in human skin^54,55^, or a ”genome set” corresponding to the complete *Homo sapiens* 3’UTR collection. To decrease the number of false positives we only considered target mRNAs predicted by all three tools and, in addition, used the control midgut set for subtraction (i.e., mRNA targets of both saliva and midgut miRNAs were discarded). This way we ended up with a “skin list” of 2011 and a “genome list” of 7188 predicted targets of the saliva miRNA set. These two lists were used to search for enriched categories by the WebGestalt^56^ tool. Using the skin list, the Gene Ontology (GO) term showing the highest enrichment ratio (ER 2.74) was the “B cell receptor signaling pathway” (GO:0050853, Supplementary Figure S5), with 16 genes targeted by the top 4 miRNAs from *Ae. aegypti* saliva. Even though the FDR value was above 0.05 (*p* value 0.000082, FDR 0.11), this result appeared especially interesting considering the crucial involvement of this signaling pathway in B cell proliferation, differentiation, and Ig production. Notably, 2 of the 16 genes were predicted targets at the same time of three of the four miRNAs (miR-1-3p, miR-14-3p and miR-1891-2-5p): (i) NFATC2 (nuclear factor of activated T cell 2, also known as NFAT1), a member of the NFAT family of transcription factors with a key role in immune responses^57^; (ii) PLCG2 (phospholipase C gamma 2), which is a transmembrane enzyme with important signaling roles in immune system cells (including B cells, natural killer cells and mast cells ) and whose mutation is involved in different conditions of immune system dysregulation^58^. The use of the whole genome list also provided some interesting sets of enriched genes, with the first and fourth Gene Ontology terms being “negative regulation of leukocyte chemotaxis” (GO:0002689; ER 3.12, *p* value 0.000241, FDR 0.021) and “Fc-epsilon receptor signaling pathway” (GO:0038095; ER 2.13, *p* value 0.000105, FDR 0.012; Supplementary Figure S6), both also pointing to the potential involvement of the most abundant *Ae. aegypti* saliva miRNAs in the manipulation of host immune, inflammatory and allergic responses.

## Discussion

Hematophagous arthropods, in their process of adaptation to blood feeding, evolved the ability to effectively manipulate vertebrate host responses to tissue damage. In this context, it is well established that mosquito salivary proteins, released with saliva at the feeding site, not only affect host haemostasis but also target inflammation and immunity, with implications for the infection and transmission of pathogens as diverse as parasites and viruses^6,11,14,19,21^. However, the non-coding RNA revolution drastically changed our understanding of eukaryotic gene expression regulation, pointing out the limits of the protein-centric view and emphasizing the crucial involvement of regulatory RNAs^59–62^. MicroRNAs are certainly the most well-known class of regulatory non-coding RNAs and it is estimated they may target over 60% of human protein-coding genes^63^. Typically, miRNAs are found within cells and are involved in the regulation of co-expressed endogenous target genes; however, they are also present in animal body fluids, where they are protected from RNase degradation by the interaction with Ago proteins or lipoproteins, or by the inclusion in extracellular vesicles^28–32^. Interestingly, exosomal microvesicles may play a role in cell–cell communication by acting as vehicles for the transfer of miRNAs (and other macromolecules), as clearly shown for exosomal miRNAs released from the adipose tissue and regulating the expression of mRNA targets in the liver^35^. This exosome-mediated miRNA transfer between cells may take place not only within the same organism but also across species, and several representative cases of involvement of small non-coding RNAs in cross-species interactions can be found in the context of the evolutionary arms race between pathogens and their hosts^64–67^. For example, the parasitic worms *H. polygyrus*, *B. malayi* and *Schistosoma japonicum*, which establish long-lasting infections of their hosts, release exosomes carrying miRNAs with immunomodulatory activity^33,34,38,68,69^. The pathogenic fungus *Beauveria bassiana* uses a miRNA-like molecule (bba-milR1) to inhibit the *Anopheles stephensi* antifungal response^70^, and viral-encoded miRNAs are known to facilitate infection and immune evasion^71^. On the other side, hosts may use their miRNAs as a defence system to fight back pathogens, as is the case for red blood cells transferring specific miRNAs to the parasite *Plasmodium*^72,73^. We have previously shown that a subset of *An. coluzzii* saliva miRNAs mimic human counterparts known to target immune and inflammatory genes, suggesting they may play a role in host manipulation^22^. More recently, Perdomo and colleagues showed that human miRNAs, ingested by *Ae. aegypti* during blood feeding, may be transferred from midgut to fat body where they can target and modulate mosquito genes^74^. These observations support a scenario where miRNAs may participate bidirectionally to the complex interactions between mosquito vectors and their vertebrate hosts. In this study, to get further insights into small non-coding RNAs involvement in vector-pathogen-host interactions, we analysed small RNA fractions from saliva and salivary glands of uninfected and CHIKV-infected *Ae. aegypti*.

An unusual finding coming out from the initial size distribution analysis of sequencing reads was the presence of a very abundant 26 nt tRF originating from the 5’-end of the Gly-GCC tRNA (Gly-GCC 5tRF). Similar tRFs are found in small RNA data sets from a large variety of organisms, although the pronounced 26 nt peaks reported here are uncommon, at least in insects small RNA-seq studies. These tRNA-derived fragments originate by non-random cleavage of pre-tRNAs or mature tRNAs, and can be grouped into different subclasses according to cleavage site and length^42^. Their regulatory function is still poorly understood but they seem to act at the transcriptional or post-transcriptional level and may target specific RNAs by a miRNA-like mechanism of action^42,75,76^. Several tRFs have been previously described in *Ae. aegypti* by Eng and collaborators, who reported an abundant 3-Pre Gly-GCC tRNA (deriving from the 3’-end of the Gly-GCC tRNA precursor) which was differentially expressed between sexes, developmental stages and following blood feeding^77^. Notably, this tRF was different from the one reported here, which originates from the mature form and encompasses the 5’-end of the tRNA; moreover, our very abundant Gly-GCC 5tRF was not among the other 55 different tRFs reported by Eng and colleagues^77^. We do not have a good explanation for this discrepancy, that may be linked to a tissue-specific high-level expression in salivary glands/saliva or to different experimental conditions. Interestingly, an identical Gly-GCC 5tRF was found in secretions and salivary glands from maggots of the green-bottle blowfly *Lucilia sericata*^78^, and a Gly-GCC 5tRF was found to be induced in mammalian endothelial cells after rickettsial infection and suggested/predicted to target genes involved in infection/inflammatory response and autophagy^79^. A deeper analysis of this 26 nt Gly-GCC 5tRF from *Ae. aegypti* is beyond the scope of this study; however, its abundance and enrichment in saliva as compared to salivary glands (FC = 4.18, *p* = 0.004, FDR = 0.039) may imply a potential functional role and deserve further investigation.

The alignment of reads from the infected samples to the CHIKV genome and antigenome provided evidence that CHIKV infection activates both the siRNA and the piRNA anti-viral pathways in *Ae. aegypti* salivary glands. In fact, the abundant 21 nt RNAs, with balanced mapping to sense and antisense strands of the viral genome, are fully compatible with vsiRNAs produced by Dcr2 activity on long dsRNAs of CHIKV origin^43,46^. These findings are common in small RNA-seq studies on *Aedes* mosquitoes (or cell lines) infected with CHIKV or other arboviruses^44^. Moreover, reads 26–30 nt in length carried distinctive signatures of their origin by the ping-pong mechanism, indicating they represent piRNA-like small RNAs of CHIKV origin, also generically named as vpiRNAs. piRNAs are commonly enriched in the germline and reproductive tissues of different animals, from drosophila to mammals, where they protect genome integrity by silencing transposable elements^80,81^. The piRNA pathway is not involved in *Drosophila* anti-viral defense^82^ but plays a relevant role in response to viral infection in mosquitoes, where the accumulation of vpiRNAs following infection by different arboviruses has been previously reported both in mosquito cell lines and in adult somatic tissues^43,46,83–87^. To the best of our knowledge there is only one study where activation of the siRNA and piRNA antiviral pathways has been analysed in the salivary glands of an arbovirus-infected mosquito. In this case WNV-specific vsiRNAs but no vpiRNAs could be revealed in the salivary glands of *Culex quinquefasciatus* infected by the West Nile virus^88^. Therefore, this represents the first report showing that *Ae. aegypti* mosquitoes, in response to chikungunya virus infection, mount in the salivary glands an anti-viral response with activation of both the siRNA and the piRNA pathways.

We report here a higher number of miRNAs in *Ae. aegypti* saliva (136 known, 19 novel) as compared to a previous study (103)^40^. This is likely due to the increased sequencing deepness (~ 2.5 vs ~ 0.6 million reads from saliva samples mapping to mature miRNAs) and, accordingly, most of the additional miRNAs were of low or very low abundance. However, the most relevant difference of our study consisted in (i) the use of triplicates and (ii) the analysis of both saliva and salivary glands. This experimental design allowed for the comparison of these two samples and for the evaluation of miRNA modulation following CHIKV infection with robust statistical support. In this context, infection-driven changes of salivary gland miRNA content may be an indication of organ-specific viral-dependent manipulation of the mosquito vector. On the other side, modulation of saliva miRNA content would change the composition of the miRNA cocktail injected into the vertebrate host during blood feeding.

We found that CHIKV infection did not induce significant changes of miRNA expression profile in *Ae. aegypti* salivary glands. This came as a surprise to us since most previous investigations, with rare exceptions^84,89^, reported an alteration of *Aedes* miRNA repertoires following infection by dengue^90–94^, Zika^86^, chikungunya^40,95,96^ or Ross River^53^ viruses. We are not in the condition to provide an unquestionable explanation for this discrepancy, however most of these experiments were in cultured mosquito cell lines, with in vivo studies performed on whole adult mosquitoes^86,91^, midgut^53,93,94^ or fat body^53^. Modulation of salivary gland miRNA expression profile was reported in the tick *Ixodes scapularis* infected by the Powassan virus^97^, but to our knowledge the effect of arboviral infection on miRNA content of mosquito salivary glands has never been analysed before. It is possible that arboviral-induced miRNA changes in mosquitoes may vary depending on the specific organ and/or phase of infection. Accordingly, minimal variation of miRNA abundance was found at late stages of *Ae. aegypti* infection by the Ross River virus (midgut and fat body) or by the Zika virus (whole mosquitoes) as compared to the earlier stages of infection^53,86^.

As far as mosquito saliva is concerned, changes in abundance of a large number of miRNAs (60 up-regulated and 15 down-regulated; |log2(FC)| > 1) were previously reported in *Ae. aegypti* and *Ae. albopictus* saliva following CHIKV infection^40^. We only found 5 miRNAs differentially expressed (2 up-regulated and 3 down-regulated; |log2(FC)| > 1 and FDR < 0.05) in infected saliva; however, because of the FDR values just below the threshold for significance, the low number of mapping reads, and the limited consistency between replicates, we considered this finding as unlikely to be of biological relevance. Perhaps the diverse experimental conditions may account for the marked difference between the two studies. First, we used an infectious blood meal and analysed saliva at 14 days post-infection (dpi) as compared to thoracic inoculation and collection of saliva at 10 dpi. It is known that the route of infection, local versus systemic, may affect innate immune responses in the mosquito^98,99^, and this may be one reason behind the different observations. Second, and more importantly, taking advantage of biological triplicates, we made use of specific software and robust statistical evaluation as opposed to the absence of replicates and simple FC calculation by the CPM ratio infected/uninfected. We believe this is the main reason for the discrepancy. Consistently, if we also calculate FC according to CPM values (without setting FDR < 0.05 as a threshold for inclusion) we also see many changes in miRNA abundance following infection (49 up-regulated and 45 down-regulated). Overall, according to the above considerations we conclude that, at least in our experimental conditions, CHIKV infection does not induce significant changes of miRNA content neither in *Ae. aegypti* salivary glands or saliva.

Our experimental setup also allowed to compare saliva to salivary glands, which highlighted the asymmetrical miRNA distribution, with groups of miRNAs preferentially enriched in *Ae. aegypti* saliva or selectively retained in salivary glands. Similar observations were previously made in the mosquito *An. coluzzii*^22^ and in the tick *I. ricinus*^23^, where saliva-enriched miRNAs carried an excess of non-templated 3’-end uridylation, suggesting their likely exosomal origin^100^. We searched *Ae. aegypti* saliva-enriched miRNAs for non-templated 3’-end uridylation and for specific EXOmotifs^101^ but did not find any molecular signature of their possible loading into exosomes. Anyhow, independently from the possible inclusion within exosomal microvesicles and the specific mechanism, distinct miRNA subsets are selectively sorted and secreted into *Ae. aegypti* saliva, with very limited impact by CHIKV infection. Noteworthy, this selective enrichment and saliva miRNA composition appeared very well conserved among the evolutionary distant *Anopheles* and *Aedes* species, who diverged around 150 million years ago, and show a remarkable degree of conservation also with very distantly related BFA as ticks (Table 3). This evolutionary conservation points to a likely physiological function suggesting a scenario in which miRNAs, injected into vertebrate host with saliva during blood feeding, may contribute to host manipulation and vector–host–pathogen interactions. These observations raise obvious questions regarding the potential mRNA targets at the biting site. *Aedes aegypti* mosquitoes, as previously reported for *An. coluzzi*^22^, carry in their saliva miRNAs identical to human miRNAs (Table 2). Most of these miRNAs (specifically aae-miR-1-3p/ hsa-miR-1-3p, aae-miR-7-5p/hsa-miR-7-5p, aae-miR-34-5p/hsa-miR-34c-5p, aae-let7-5p/hsa-let-7a-5p, aae-miR-8-3p/hsa-miR-141-3p, aae-miR-184-3p/hsa-miR-184, aae-miR-100-5p/hsa-miR-100-5p, aae-miR-8-5p/hsa-miR-200b-5p, aae-miR-125-5p/hsa-miR-125b-5p) were experimentally shown to target human mRNAs involved in immune and inflammatory responses, as for example transcripts coding for cytokines, chemokines, chemokine receptors and transcription factors with key roles in the NF-kB (Nuclear Factor kappa B) or TLRs (Toll-Like Receptors) signaling pathways (see Supplementary Tables S2 and S3^22^ for additional details). Besides this evidence, which comes from experimentally validated targets, we also used available prediction tools to search for potential targets of miRNAs from *Ae. aegypti* saliva. Having in mind that miRNAs need to reach a certain abundance to mediate significant target suppression^102^, and to reduce the background noise often generated by prediction tools, we used the four most abundant saliva miRNAs and subtracted those targets predicted using a control miRNA set. We believe that the results of over-representation analysis support the possible involvement of miRNAs from *Ae. aegypti* saliva in manipulation of host immune, inflammatory and allergic responses. In this respect the enriched GO terms “B cell receptor signaling pathway” and “Fc-epsilon receptor signaling pathway” emerging from prediction analysis appear especially meaningful from the biological point of view. The presence of miRNAs identical to human miRNAs in saliva of mosquitoes and ticks, and in exosomes released by parasitic nematodes, is intriguing and raises the fascinating hypothesis of a common strategy for host manipulation. Indeed, the use of miRNAs that mimic host miRNAs would allow to exploit a conserved network of host miRNA target sites, as previously suggested for virus-encoded miRNAs^71^. The evolutionary advantage of such a strategy for a mosquito, who gets its blood meal within a time frame of seconds or minutes, is rather obscure considering the miRNAs mechanism of action. However, saliva is a complex cocktail and carries both proteins and miRNAs, who may act synergistically. In this scenario mosquito salivary proteins, injected into the host skin during feeding, may provide an immediate reward to the individual through their fast-acting biochemical and pharmacological properties^6^. On the other side the slower-acting miRNAs may provide a later advantage to the population, and therefore to the species, modulating host immune and inflammatory responses. This may result, for example, in the downregulation of anti-saliva antibody responses and/or of allergic reactions and would agree with the results of target prediction analysis. Reducing the anti-saliva humoral response may be beneficial because antibody-mediated inactivation of salivary protein functions decreases blood feeding efficiency with detrimental effects at population and species level. On the other side, strong allergic reactions to saliva may set in the host a high alert status that may be dangerous for a vector in search of its host.

We would like to remind the reader that our experimental setup involved the use of artificial membranes and rabbit blood rather than feeding through skin on human blood. We cannot rule out its possible influence on miRNA expression profiles, especially early after feeding. However, considering that our samples were collected two weeks after feeding, we do not expect that these departures from natural *Ae. aegypti* blood acquisition may significantly affect miRNA content of mosquito salivary glands and saliva. Further studies and experimental validations will be certainly needed to clarify the possible contribution of miRNAs from mosquito saliva to host manipulation and perhaps pathogen transmission. This is certainly going to be challenging considering the intricacy and the difficulty of working at the vector-host interface. Nevertheless, we believe that the experimental evidence reported here offers novel point of views of the complex interactions between mosquitoes, vertebrates and vector-borne pathogens and may represent a useful starting point for future investigations on the role of mosquito miRNAs in pathogens transmission.

## Materials and methods

### Mosquito rearing and *Ae. aegypti* infection

*Aedes aegypti* mosquitoes PAEA strain originally collected at Paea (Tahiti, French Polynesia; colonized since 1994) were reared under standard insectary conditions (28 ± 1 °C, 70% relative humidity, 12:12 h light:dark photoperiod). The CHIKV 06-21 strain (Reunion Island, Virus Pathogen Resource AM258992) was used for experimental infections, which were performed in the BSL-3 laboratory at Institute Pasteur (Paris, France). Typically, adult female mosquitoes 10-days-old were starved for 24 h before feeding with an Hemotek® membrane feeding system for 30 min at 37 °C. The infectious blood meal was composed by 1.4 ml washed defibrinated rabbit blood, 0.7 ml viral suspension and 5 mM ATP. Virus titers of blood meals were at 10^7^ ffu/ml. In non-infectious blood meal, viral suspension was replaced by cell culture medium. Fully engorged mosquitoes were selected and kept in the insectarium of BSL-3 at 28 °C and 70% relative humidity until analysed. The same experimental procedure was applied to infected and uninfected mosquitoes, with the only difference being the presence in the artificial blood meal of the chikungunya virus as described above.

### Mosquito salivation and salivary gland dissection

For each experimental group, saliva was collected from 150 mosquitoes and salivary glands were dissected from 80 mosquitoes, 14 days after the blood meal (either challenged or unchallenged). Experiments were done in triplicate. Typically, under the standard protocols used in the insectary of Pasteur Institute, at 14 days post infection the dissemination rates of CHIKV 06.21 in the *Ae. aegypti* PAEA strain range from 90 to 98%. At the same time, salivary glands are infected (as determined by immunofluorescence assays) and the number of viral RNA copies per gland is around 10^3^ or more (ABF, personal communication; see Vazeille M et al. 2007 for additional experimental details). For saliva collection, mosquitoes were cold-anesthetized and, after removing wings and legs, the proboscis was inserted into a 10 μl tip filled with 1 μl of PBS. Mosquitoes were left to salivate for 30 min, then the saliva-containing PBS was expelled from the tips into a 1.5 ml collection tube containing QIAzol lysis reagent (Qiagen 79,306) at a ratio 5:1 (V:V). Salivary glands were dissected in PBS and transferred into 1.5 ml tubes kept on ice and containing QIAzol lysis reagent. Typically, 80 salivary glands pairs were collected in 700 μl of QIAzol lysis reagent and mechanically homogenized. Both saliva and salivary gland samples were stored at -80 °C until RNA extraction.

### RNA extraction, library construction and Illumina sequencing

Small RNA fractions (< 200 nt) were extracted from uninfected and CHIKV-infected *Ae. aegypti* saliva and salivary glands using the miRNeasy Serum/Plasma kit (Qiagen 217184) and the miRNeasy Micro kit (Qiagen 217084), respectively, according to manufacturer instructions. RNAs were extracted from three biological replicates of saliva, collected from 150 adult female mosquitoes, or from 80 adult female salivary glands. RNA size distribution and concentration were assessed using the RNA Pico 6000 Assay Kit for the Bioanalyzer 2100 system (Agilent Technologies). Small RNA libraries were prepared from 1 ng of RNA using the NEXTflex Small RNA-seq Kit v3 (Perkin Elmer) according to manufacturer instructions. The size distribution of the libraries was assessed on a Bioanalyzer with a DNA High Sensitivity kit (Agilent Technologies); concentration was measured using a Qubit® DNA High Sensitivity kit in a Qubit® 2.0 Fluorometer (Life Technologies). Libraries that passed the QC step were pooled in equimolar amount and final pool was purified by the SPRIselect beads (Beckman Coulter) with a 1.3 × ratio. Fifty base pair, single end sequencing was performed on an Illumina HiSeq2500 platform. RNA quality control and libraries preparation were performed by the EMBL Genomic Core Facility (EMBL, Heidelberg, DE).

### Reads mapping

Raw reads were quality control checked by FastQC^103^ and then trimmed using cutadapt 1.9.1^104^ to remove 3’ adapters and discard reads shorter than 14 nucleotides. Processed reads from each sample were aligned to the *Ae. aegypti* AaegL5 genome assembly (Liverpool AGWG strain, VectorBase^105^) using the aligner tool Bowtie^106^ (-n 0 -l 18 -e 80). Reads not mapping to AaegL5 where aligned (-n 0 -l 18 -e 80 -norc) to the CHIKV RNA genome and antigenome (strain 06-21, Reunion isolate, Virus Pathogen Resource AM258992). Reads mapping to AaegL5 were then aligned (-n 0 -l 18 -e 80 –norc) to a collection of *Ae. aegypti* rRNA sequences obtained from VectorBase by the BioMart tool^107^. Reads unaligned to rRNA were used to analyse the size distribution reported in Fig. 1 and then mapped (-n 0 -l 18 -a –best –strata -e 80 -norc) to a list composed of 327 miRNA precursors plus other non-coding RNAs from *Ae. aegypti*. Reads aligning to this list of hairpins and ncRNAs were used for correlation analysis, mapping to mature miRNAs and differential expression analysis; unaligned reads were sequentially mapped, using the same parameters as above, to collections of *Ae. aegypti* lncRNAs, transcripts (AaegL5.2) and repeats retrieved from VectorBase.

The list of 327 miRNA precursors included 144 previously known *Ae. aegypti* miRNA precursors plus 183 hairpins predicted by miRDeep*^108^. The known miRNA precursors included 125 hairpins retrieved from miRBase v22^109^ and 19 additional hairpins found in previous studies^40,110,111^. The other *Ae. aegypti* small ncRNAs were retrieved from VectorBase using the BioMart tool and included tRNAs, snRNAs, snoRNAs, RNase P, RNase MRP, arthropod 7SK and SRP RNA. The collection of mature miRNAs consisted of 425 miRNAs (5p + 3p): 161 retrieved from miRBase v22, 81 from previous studies^40,110,111^ and 183 predicted by miRDeep*. Lists of precursors and mature miRNAs are provided in Supplementary File S1.

### Prediction of putative novel *Ae. aegypti* miRNAs

The miRDeep* tool^108^, which allows for miRNA prediction from RNA sequencing data, was used to identify putative novel *Ae. aegypti* miRNAs. Briefly, reads mapping to AaegL5 were first depleted of those aligning to rRNAs and then mapped to a list of 144 known *Ae. aegypti* miRNA precursors and other ncRNAs (see above). Unaligned reads from the 12 libraries were concatenated, subtracted of repeats, and then used as input for miRDeep*. After filtering the predictions using a miRDeep* score threshold equal to 0, a total of 183 miRNA precursors were retained as putative novel *Ae. aegypti* miRNAs. This way lists of 327 miRNA precursors and 425 mature miRNAs were assembled for *Ae. aegypti* (Supplementary File S1) and used for the following analyses.

### Quantification and differential expression

Read counts for each *Ae. aegypti* small RNA library were computed from SAM files using a Python custom script. Reads with multiple highest score mappings were discarded (i.e., multi-mapping reads were not considered when having the same highest score). Expression values were calculated as CPM (Counts Per Million of mapped reads, that is the number of reads mapping on a feature divided by the total number of mapped reads and multiplied by one million) and used for sample clustering. Reads mapping to precursor miRNAs were assigned to mature miRNAs based on their mapping position; overhangs of maximum 3 nucleotides for each side of the mature form were tolerated. Differential expression analysis of mature miRNAs with expression equal to or greater than 1 CPM in at least three samples was performed using glmFIT and glmLRT functions provided by the edgeR software package^47,48^. Log2 Fold change (FC) and false discovery rates (FDR) were calculated to provide statistical validation. Proportions of reads mapping to miRNA hairpins were compared by the Chi-square test with Yates’ correction. Custom scripts used to count reads mapping to ncRNAs, hairpins and mature miRNAs are included as a zipped folder named Supplementary Custom Script.

### Target prediction

Human genes putatively targeted by miRNAs from *Ae. aegypti* saliva were predicted taking advantage of miRNAconsTarget; this tool allows for the parallel use of the prediction algorithms TargetSpy^50^, miRanda^51^ and PITA^52^ and is available at the sRNAtoolbox^112^ website (https://arn.ugr.es/srnatoolbox/). The miRNAs from *Ae. aegypti* saliva aae-miR-14-3p, aae-miR-1891-2-5p, aae-miR-1-3p and aae-miR-276-1-3p were used as query; these were the four most abundant miRNAs both in the saliva of uninfected and CHIKV-infected mosquitoes (CPM range 89,709–241,459; Supplementary file S3). As a control set the miRNAs from *Ae. aegypti* midgut aae-miR-281-3p, aae-miR-100-5p, aae-miR-184-3p and aae-miR-283-5p were employed; these were the four most abundant miRNAs in *Ae. aegypti* midgut as determined by retrieving a small RNA dataset from adult *Ae. aegypti* midgut deposited by Sinclair and collaborators^53^ at the Sequence Read Archive (SRA, https://www.ncbi.nlm.nih.gov/sra; BioProject PRJNA635740; SRR11870698, SRR11870700 and SRR11870701). Briefly, after trimming, quality filtering and mapping to our collection of mature miRNAs, data from this triplicate sample were used to compile a list of midgut *Ae. aegypti* miRNAs. These top four midgut miRNAs (CPM range 69,692–162,948) were also present in our saliva samples but at lower abundance (rank 13–90). Independent predictions were run with the saliva and midgut miRNAs, searching for targets in two different sets of 3’UTR: (i) a “skin set”, composed of 15,668 3'UTRs from transcripts expressed in human skin^54,55^ (downloaded from Ensembl^113^ using the BioMart tool^114^), and (ii) a “genome set” represented by the *Homo sapiens* 3’UTRs collection available within the miRNAconsTarget tool. To reduce the background noise and increase specificity, only miRNA-mRNA interactions predicted by all three tools (TargetSpy, miRanda and PITA) were taken into consideration and, in addition, putative targets of midgut miRNAs were subtracted from the list of targets of saliva miRNAs. This way two lists of putative targets of the saliva miRNAs were obtained: a “skin list” and a “genome list”. Enrichment analysis was performed taking advantage of the WebGestalt tool^56^ (WEB-based Gene SeT Analysis Toolkit, http://www.webgestalt.org/) using the “skin list” and “genome list” as queries and the “skin set” and “genome set” as references.

### Ethical statement

Experimental protocols reported in this study were approved by the animal experimentation Ethics Committee of the Institute Pasteur and registered under the reference APAFIS (Autorisation de Projet utilisant des Animaux à des FIns Scientifiques) #6573-201606l412077987 v2. Animals were housed in the Institut Pasteur animal facilities (Paris) accredited by the French Ministry of Agriculture. Work on animals was performed in compliance with French and European regulations on care and protection of laboratory animals (EC Directive 2010/63, French Law 2013-118, February 6th, 2013). No experimental research on humans or use of human tissues is carried out in this study. All methods are reported in accordance with ARRIVE guidelines (https://arriveguidelines.org).

## Supplementary Information

Supplementary Information 1.

Supplementary Information 2.

Supplementary Information 3.

Supplementary Information 4.

Supplementary Information 5.

Supplementary Information 6.

## Data Availability

The small RNA-Seq datasets generated and analysed during the current study have been deposited in NCBI’s Gene Expression Omnibus^115^ and are accessible through Geo Series accession number GSE174512 (https://www.ncbi.nlm.nih.gov/geo/query/acc.cgi?acc=GSE174512). Other data generated during this study have been included as Supplementary Information.
