# Supplementary material for: MicroRNAs and other small RNAs in Aedes aegypti saliva and salivary glands following chikungunya virus infection
Source: Sci Rep. 2022 Jun 9;12:9536. doi: 10.1038/s41598-022-13780-3 (PMC9184468; doi:10.1038/s41598-022-13780-3)
Supplement: Supplementary file 5 — Supplementary Information 5. [file 41598_2022_13780_MOESM5_ESM.pdf]

Supplementary Figures

MicroRNAs and other small RNAs in *Aedes aegypti* saliva and salivary glands following chikungunya virus infection

Carmine Fiorillo[1†], Pei-Shi Yen[2†], Alessio Colantoni[3], Marina Mariconti[2], Nayara Azevedo[4], Fabrizio Lombardo[1], Anna-Bella Failloux[2], Bruno Arcà[1*]

1. *Department of Public Health and Infectious Diseases, "Sapienza" University, Piazzale Aldo Moro 5 – 00185 Rome, Italy.*
2. *Arboviruses and Insect Vectors Unit, Institute Pasteur, 25 rue Dr. Roux, CEDEX 15, 75724 Paris, France.*
3. *Department of Biology and Biotechnology, "Sapienza" University, Piazzale Aldo Moro 5 – 00185 Rome, Italy.*
4. *Genomics Core Facility, European Molecular Biology Laboratory, Meyerhofstrasse 1, 69117 Heidelberg, Germany.*

† These authors contributed equally to this work

***Corresponding author**:

Bruno Arcà, Department of Public Health and Infectious Diseases – Division of Parasitology, Sapienza University, Piazzale Aldo Moro 5, 00185 Rome, Italy. Tel: +39 06 4991 4413. E-mail: bruno.arca@uniroma1.it

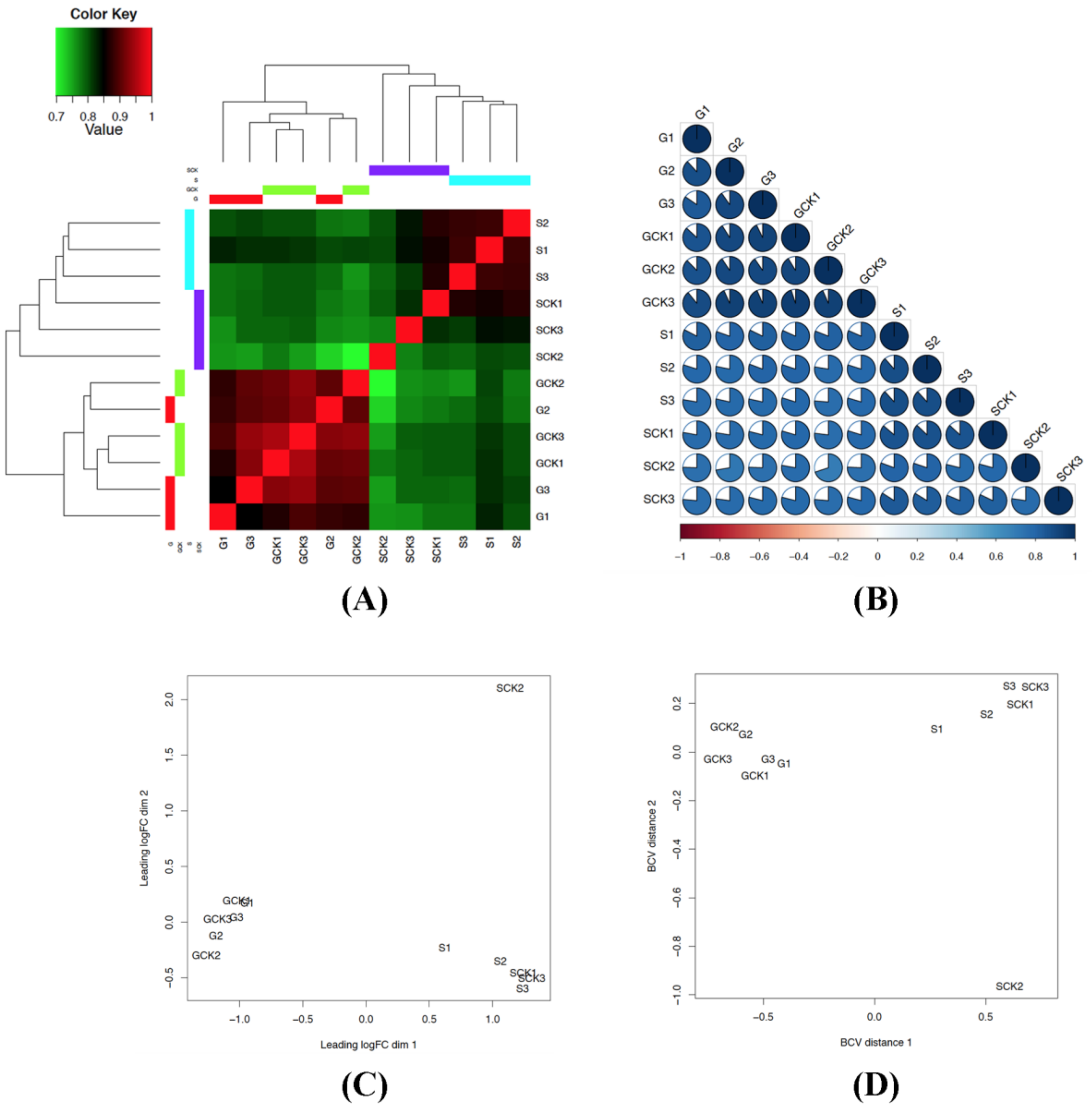

Supplementary Figure S1. Correlation among replicates and samples analyzed in this study. (**A**) Small RNAs expression-based clustering of the *Ae. aegypti* G (red bars), GCK (green bars), S (light blue bars) and SCK (purple bars) samples. Cells represent color-coded Pearson correlation coefficients measuring the similarity of small RNA expression profiles between two samples. Such correlation values were also used for the hierarchical clustering of samples. Expression values used to build the matrix were calculated as log2-transformed CPM values. Only small non-coding RNAs with CPM ≥ 1 in at least three samples were used. The heatmap was drawn using custom R code. (**B**) Pair-wise correlation analysis of the 12 *Ae. aegypti* samples using the corrplot package in R (ver. 4.0.3). All samples show positive correlation with each other (blue color), with color intensity and size proportional to Spearman's correlation coefficients (S, 0.88-0.89; G, 0.85-0.90; SCK, 0.77-0.82; GCK, 0.91-0.95). (**C**-**D**) Multidimensional scaling plots based on Fold Change (C) and Biological Coefficient of Variation (D). Plots were generated using the plotMDS function implemented in the edgeR software package. Only small non-coding RNAs with CPM ≥ 1 in at least three samples were used.

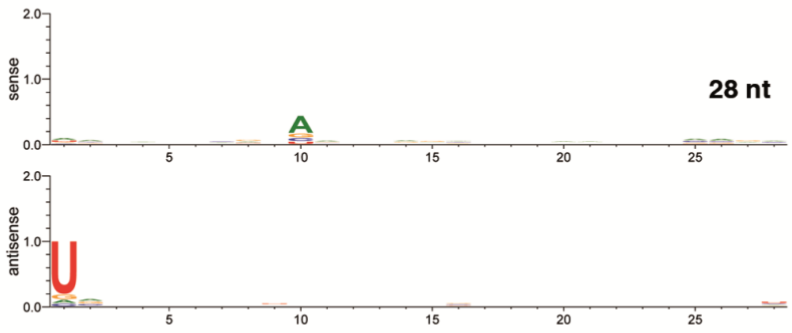

Supplementary Figure S2. Signatures of ping-pong amplification in 28 nt piRNA-like viral small RNAs from the salivary glands of CHIKV-infected *Aedes aegypti*. Nucleotide analysis of 28 nt fractions from the GCK samples mapping to the plus (upper panel) and minus (lower panel) strands of the CHIKV genome. The A10 bias in reads mapping to the sense strand, and the U1 bias in those mapping to the antisense strand, are highlighted by the sequence logos generated by the web-based application WebLogo3 (http://weblogo.threeplusone.com/).

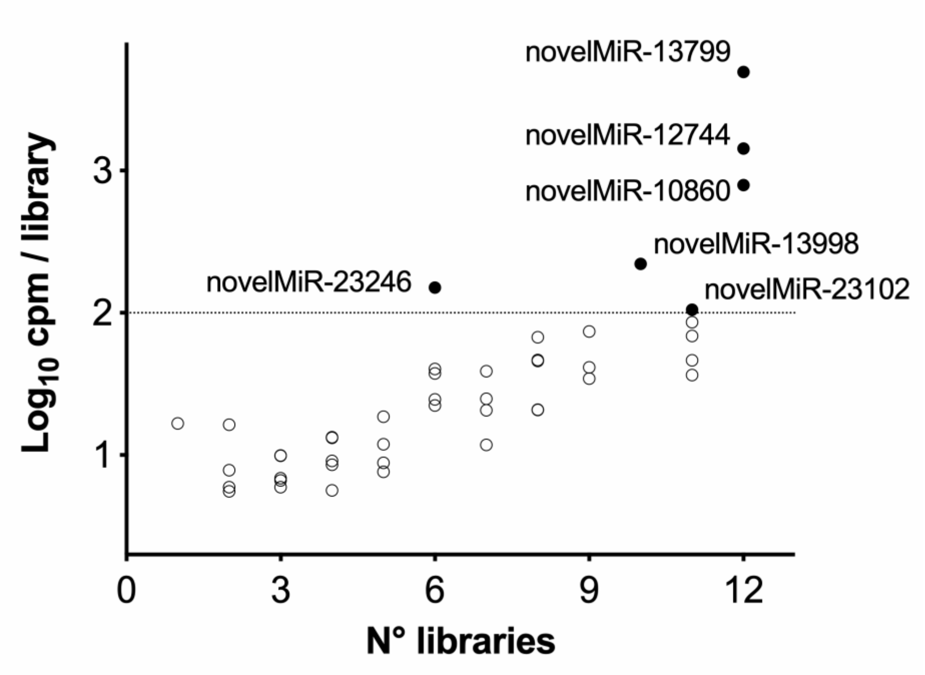

Supplementary Figure S3. Abundance of the forty-five predicted miRNAs in the twelve libraries. For each of the putative 45 novel *Ae. aegypti* miRNAs predicted by the miRDeep* tool, the number of libraries with CPM ≥ 3 and the mean CPM per library are reported. The six miRNAs with > 100 CPM/library (dashed line) are shown with filled circles.

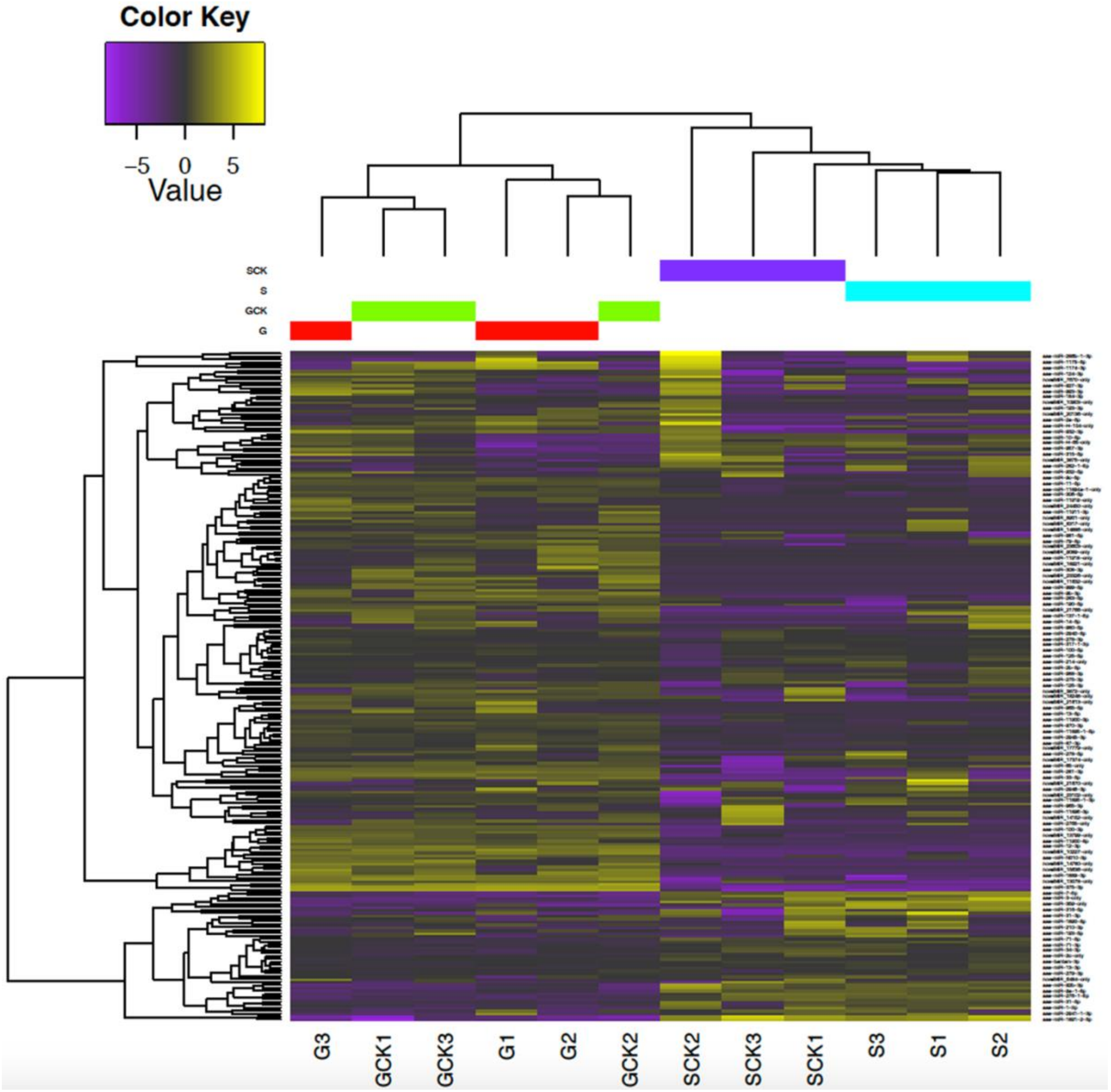

Supplementary Figure S4. Mature miRNA expression profiles. Mature miRNA expression heatmap and hierarchical clustering of G (red bar), GCK (green bar), S (light blue bar) and SCK (purple bar) samples. Cells correspond to mean-centered log2-transformed color-coded CPM values. Only mature miRNAs with ≥ 1 CPM in at least three samples were used. The heatmap was drawn using custom R code.

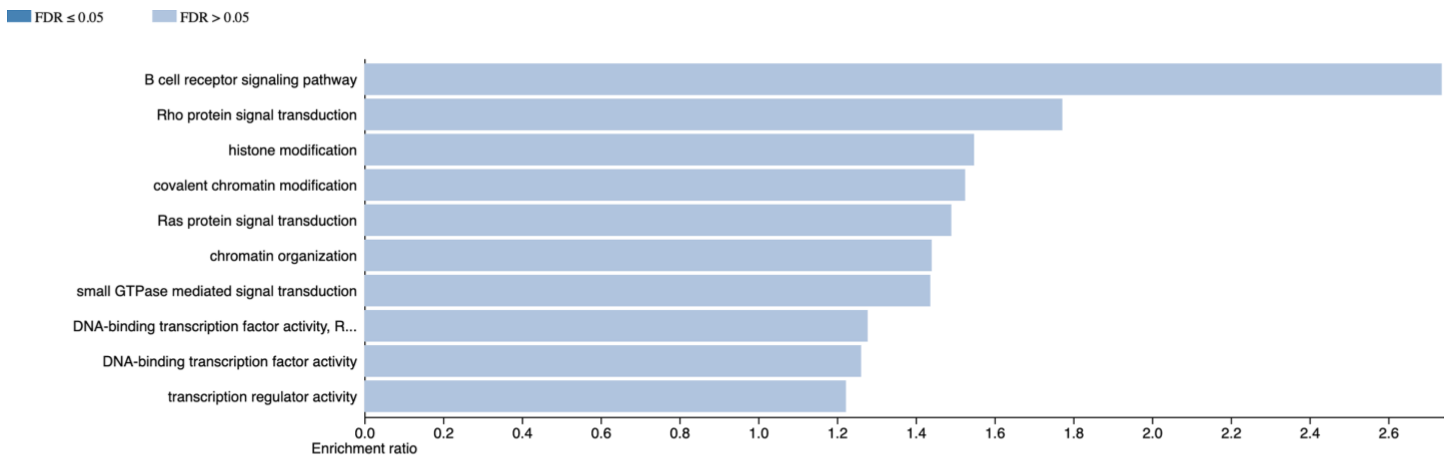

Gene Set	Description	Size	Expect	Ratio	P Value	FDR
GO:0050853	B cell receptor signaling pathway	41	5.8483	2.7359	0.000082231	0.11339
GO:0007266	Rho protein signal transduction	178	25.39	1.7723	0.000065967	0.11339
GO:0016570	histone modification	403	57.484	1.5483	0.000011322	0.067641
GO:0016569	covalent chromatin modification	409	58.34	1.5255	0.000020642	0.082219
GO:0007265	Ras protein signal transduction	381	54.346	1.4904	0.00011027	0.11339
GO:0006325	chromatin organization	618	88.152	1.4407	8.1141E-06	0.067641
GO:0007264	small GTPase mediated signal transduction	483	68.896	1.437	0.000089208	0.11339
GO:0000981	DNA-binding transcription factor activity, RNA polymerase II-specific	1152	164.32	1.278	0.000054527	0.11339
GO:0003700	DNA-binding transcription factor activity	1245	177.59	1.2613	0.000070992	0.11339
GO:0140110	transcription regulator activity	1548	220.81	1.2228	0.00011558	0.11339

Supplementary Figure S5. Results of functional enrichment analysis using as query the list of predicted targets among genes expressed in human skin. Over-Representation Analysis was performed using the WebGestalt toolkit. Bars show the enrichment ratio whereas Gene Ontology (GO) terms (Biological Process, Cellular Component, Molecular Function) and/or pathways (KEGG, Panther, Wikipathway) are reported on the left. The top 10 results are shown.

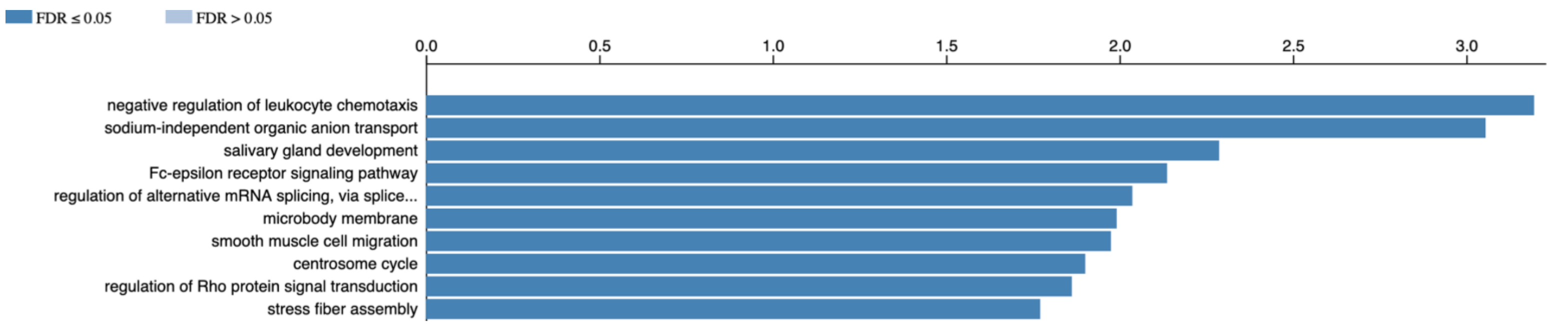

Gene Set	Description	Size	Expect	Ratio	P Value	FDR
GO:0002689	**negative regulation of leukocyte chemotaxis**	17	3.1296	3.1953	0.00024125	0.021817
GO:0043252	sodium-independent organic anion transport	16	2.9455	3.0555	0.0007842	0.045703
GO:0007431	salivary gland development	38	6.9956	2.2871	0.00060481	0.041252
GO:0038095	**Fc-epsilon receptor signaling pathway**	61	11.23	2.1372	0.00010565	0.012152
GO:0000381	regulation of alternative mRNA splicing, via spliceosome	64	11.782	2.037	0.00025204	0.022331
GO:0031903	microbody membrane	60	11.046	1.9917	0.0006441	0.042661
GO:0014909	smooth muscle cell migration	66	12.15	1.9753	0.00042977	0.034363
GO:0007098	centrosome cycle	120	22.091	1.9012	0.000011354	0.0026588
GO:0035023	regulation of Rho protein signal transduction	140	25.773	1.8624	5.3493E-06	0.0016305
GO:0043149	stress fiber assembly	92	16.937	1.7713	0.00076229	0.045306

Supplementary Figure S6. Results of functional enrichment analysis using as query the list of predicted targets among the entire human gene set. Over-Representation Analysis was performed using the WebGestalt toolkit. Bars show the enrichment ratio whereas Gene Ontology (GO) terms (Biological Process, Cellular Component, Molecular Function) and/or pathways (KEGG, Panther, Wikipathway) are reported on the left. Only the top 10 enriched GO terms and/or pathways with FDR < 0.05 are reported.
